# Unilateral boundary time series forecasting

**DOI:** 10.3389/fdata.2024.1376023

**Published:** 2024-06-05

**Authors:** Chao-Min Chang, Cheng-Te Li, Shou-De Lin

**Affiliations:** ^1^Department of Computer Science and Information Engineering, National Taiwan University, Taipei, Taiwan; ^2^Department of Computer Science and Information Engineering, National Cheng Kung University, Tainan, Taiwan

**Keywords:** time series forecasting, unilateral boundary, asymmetric loss function, feature reconstruction, dual model structure

## Abstract

Time series forecasting is an essential tool across numerous domains, yet traditional models often falter when faced with unilateral boundary conditions, where data is systematically overestimated or underestimated. This paper introduces a novel approach to the task of unilateral boundary time series forecasting. Our research bridges the gap in existing methods by proposing a specialized framework to accurately forecast within these skewed datasets. The cornerstone of our approach is the unilateral mean square error (UMSE), an asymmetric loss function that strategically addresses underestimation biases in training data, improving the precision of forecasts. We further enhance model performance through the implementation of a dual model structure that processes underestimated and accurately estimated data points separately, allowing for a nuanced analysis of the data trends. Additionally, feature reconstruction is employed to recapture obscured dynamics, ensuring a comprehensive understanding of the data. We demonstrate the effectiveness of our methods through extensive experimentation with LightGBM and GRU models across diverse datasets, showcasing superior accuracy and robustness in comparison to traditional models and existing methods. Our findings not only validate the efficacy of our approach but also reveal its model-independence and broad applicability. This work lays the groundwork for future research in this domain, opening new avenues for sophisticated analytical models in various industries where precise time series forecasting is crucial.

## 1 Introduction

Unilateral boundary time series forecasting is a nuanced and intricate domain within the field of time series analysis. This form of forecasting pertains to time series data that is characterized by the presence of only one boundary, either as a lower or an upper bound. The essence of this concept lies in the fact that time series with a lower bound tend to be partially underestimated. In simpler terms, the values represented in these series are frequently less than the actual figures, thus positioning the given time series as a lower boundary of the real data. Conversely, time series that feature an upper bound are partially overestimated, where the represented values exceed the actual data, establishing the given series as an upper boundary.

Real-world applications abound with examples of single boundary time series problems. A common instance is observed in the performance of multiple low-cost sensors. These sensors, under specific conditions, either underestimate or overestimate the actual values they are designed to measure. This phenomenon is well-documented in various studies.

- Rohan et al. conducted an in-depth investigation into the Plantower PMS1003, a low-cost *particle sensor*. Their research revealed a significant spike in particle number and mass concentrations when the relative humidity exceeded ~75% (Jayaratne et al., 2018). This situation typifies what can be termed as a *secondary* time series sample, where the deviation from accuracy—be it overestimation or underestimation—correlates with another time series.
- Another intriguing case study scrutinized the efficacy of various *rainfall* measurement instruments, including a tipping bucket rain gauge (TBRG), a weighing rain gauge (WRG), an optical rain gauge (ORG), a present weather detector (PWD), a Joss–Waldvogel disdrometer (JWD), and a 2-D video disdrometer (2DVD), to evaluate how accurately they measure rainfall and drop size distributions. Their findings were quite revealing: the PWD and 2DVD recorded higher, and the JWD lower rain rates when the rainfall intensity exceeded 20 mm/h, while the TBRG recorded higher and the WRG lower rain rates below this threshold (Liu et al., 2013). This scenario is a classic example of an output sample, where the variation in measurement is intrinsically linked to the value within the time series itself.
- Girisha et al. explored the accuracy of low-cost *soil water sensors*, namely ECH2O-5TE, Watermark 200SS, and Tensiometer model R. Their study indicated that while the ECH2O-5TE tended to overestimate soil water content, the Watermark and Tensiometer underestimated it (Ganjegunte et al., 2012). This scenario exemplifies what can be described as a random perturbation sample, where the accuracy deviation occurs in a seemingly random manner.

The potential for practical applications of unilateral boundary time series forecasting extends to a variety of fields. For instance, in the transportation sector, a taxi company might be interested in forecasting the potential number of waiting passengers in a specific area. Historically, it is more feasible for taxi companies to record data on carried passengers rather than those waiting. It is evident that the number of carried passengers underestimates the actual number of potential passengers, especially when taxi availability is low. Similarly, parking lot owners can employ this forecasting model to predict the potential number of vehicles needing parking space, using historical data on occupied spaces. Both the taxi and parking scenarios can be categorized as upper bound samples, where underestimation occurs once the values surpass a certain threshold, like the number of available taxis or parking spaces.

The challenge and opportunity in unilateral boundary time series forecasting lie in developing models capable of accurately predicting actual figures based on historically underestimated or overestimated data. This necessitates not only an understanding of the specific characteristics of the data but also an ability to discern and account for the underlying factors that contribute to the boundary conditions. The examples cited from various studies highlight the diverse contexts in which unilateral boundary time series are relevant and underscore the need for sophisticated modeling techniques to tackle this complex yet fascinating area of time series analysis.

In the aforementioned scenarios, it is notable that we possess the capability to discern instances of data underestimation or overestimation. Take, for instance, the PMS1003 low-cost particle sensor. This device tends to overestimate readings at relative humidity levels surpassing 75%. Consequently, we can assume that in historical particle time series data, instances where relative humidity exceeded 75% likely led to overestimated measurements. Conversely, consider the scenario concerning potential waiting taxi passengers. Here, the total number of taxi passengers is prone to underestimation in situations where there are minimal or no vacant taxis available. Therefore, it is reasonable to assume that in such instances, the aggregate count of taxi passengers has been underestimated, underscoring a direct correlation between taxi availability and passenger count estimation. Addressing either the lower-bounded or upper-bounded time series forecasting problem can be methodologically equivalent. By simply reversing the sign of each time series value, an upper-bounded series can be transformed into a lower-bounded one, and vice versa. This duality offers a versatile framework for model development.

Given a set of time series data characterized by unilateral boundaries, where some values are known to be either overestimated or underestimated under specific conditions, the problem of *Unilateral Boundary Time Series Forecasting* involves developing a predictive model that can accurately forecast future values by dealing with these skewed segments in the training dataset. To the best of our knowledge, there appears to be a noticeable gap in the existing literature and practical applications concerning models specifically designed for unilateral boundary time series forecasting. This particular niche in time series analysis, which deals with datasets that are characterized by either partial overestimation or underestimation, remains largely unexplored. Recognizing this void, our research endeavors to construct a comprehensive framework tailored to address this unique forecasting challenge.

Our proposed method for unilateral boundary time series forecasting is novel in novel and multifaceted, targeting three key aspects of the challenge. The first is the introduction of an asymmetric loss function, the loss function of unilateral mean square error (UMSE), tailored to address underestimated output data in training datasets. This approach significantly improves the model's accuracy by correcting bias toward underestimation. Second, we have developed a dual model structure, specifically designed to handle underestimated input data in both training and testing phases. This structure is critical for aligning the model's predictions with the actual data trends, ensuring a more accurate and unbiased forecast. Last, the technique of feature reconstruction plays a key role. It involves enhancing the actual input data to capture the underlying dynamics that may be obscured due to unilateral boundary conditions. This step is essential for a deeper and more comprehensive data analysis, leading to more accurate forecasting outcomes. Together, these strategies create a robust framework for addressing the nuances of unilateral boundary time series forecasting, ensuring precision and reliability in predictions.

To evaluate the efficacy of our proposed method, we conducted experiments utilizing Light Gradient Boosting Machine (LightGBM) (Ke et al., 2017) and Gated Recurrent Unit (GRU) (Cho et al., 2014) models. These models were chosen due to their widespread use and proven effectiveness in time series forecasting. LGBM is renowned for its efficiency and effectiveness as a tree-based model, while GRU is a celebrated recurrent neural network model. The objective of employing these models was to demonstrate the model-independence of our method and their applicability across different forecasting paradigms. Our experimentation extended to various datasets and underestimation simulation methods. This comprehensive approach was instrumental in showcasing the versatility and robustness of our methods across diverse data sets and underestimation scenarios. Additionally, we conducted thorough comparative analyses against other related methods to underscore the advancements our methods offer.

The contributions of this work are four-fold.

- Conceptually, we propose and formulate the problem of unilateral boundary time series forecasting, which is widely motivated by various real-world scenarios of time series data collection and analysis.
- Technically, we have put forth a generalized model for unilateral boundary time series forecasting, amalgamating three distinct strategies, including the loss function of unilateral mean square error, the dual model structure, and the feature reconstruction module, to address the multifaceted nature of this forecasting challenge.
- Empirically, our simulation of various underestimation scenarios, grounded in real-world applications, coupled with experiments on diverse datasets, establishes the efficacy of our method across a broad spectrum of situations and data types.
- Lastly, the successful application of our method on both tree-based and deep learning models not only reinforces their versatility but also opens avenues for their implementation across various model architectures.

The organization of the remainder of the paper is as follows. Section 2 critically reviews existing studies, highlighting the limitations in current forecasting methodologies and setting the stage for our novel contributions. In Section 3, we delineate the problem statement, defining the scope and the specific challenges of unilateral boundary time series forecasting. Section 4 is devoted to the technical exposition of our proposed method, detailing the dual model structure, the innovative asymmetric loss function, and the feature reconstruction technique that collectively enhance forecasting accuracy. Experimental setups, including the datasets and evaluation metrics used, along with a thorough analysis of the results, are presented in Section 5. This section validates the effectiveness of our approach through comparative studies with existing models. Finally, Section 6 concludes the paper with a summary of our findings, the implications for practical applications, and directions for future research.

## 2 Related work

This section provides a comprehensive review of the literature pertinent to unilateral boundary time series forecasting. We delineate various forecasting methodologies, particularly focusing on those that address distribution shifts and non-stationary time series, which share conceptual similarities with unilateral boundary conditions. Each referenced study is critically evaluated to highlight both its contributions and limitations in the context of unilateral boundary challenges. This review sets a critical foundation for the subsequent sections by clearly establishing the existing gaps that our proposed methodology aims to fill, thereby justifying the need for our novel approach. This section not only contextualizes our work within the broader field of time series forecasting but also underscores the novelty and necessity of tackling the specific problem of unilateral boundaries in forecasting models.

### 2.1 Time series forecasting with distribution shift

Time series forecasting with distribution shift refers to the prediction of future values in a series where the underlying data distribution changes over time. This is a complex issue because traditional forecasting methods often assume that past data distributions will continue into the future, which is not the case with distribution shifts. Recent advancements in this field have introduced several novel approaches. Fan et al. (2023) have developed Dish-TS, a paradigm designed to alleviate distribution shifts by considering intra-space (within input data) and inter-space (between input and output data) shifts. Their dual coefficient network framework, Dish-TS, adjusts for these shifts to enhance forecasting accuracy significantly. Duan et al. (2023) introduced Hyper TimeSeries Forecasting (HTSF), a hypernetwork-based framework that adapts to time-varying distributions and forecasts accurately under distribution shifts, using hyper layers to characterize and adjust to these shifts dynamically. Kim et al. (2021) proposed reversible instance normalization (RevIN), a method for addressing changes in statistical properties over time by symmetrically removing and restoring statistical information within a time series instance. This approach has shown to improve forecasting in various real-world datasets. Wang et al. (2023) took inspiration from Koopman theory to create the Koopman Neural Forecaster (KNF), a sequence model that gains robustness against distributional shifts by learning a linear Koopman space with neural networks. Cai et al. (2023) tackled the concept drift problem with MemDA, encoding periodicity in the data and adapting on-the-fly to changes using a meta-dynamic network, thus enhancing model generalizability across distribution changes. Lastly, Chen et al. (2023) focused on calibrating Transformers for time series forecasting, introducing a method to detect and adapt to context-driven distribution shifts (CDS) using a residual-based detector and a sample-level contextualized adapter.

Time series forecasting with distribution shift is distinctly different from unilateral boundary time series forecasting. While the former deals with the entire data distribution changing over time, the latter specifically addresses scenarios where the series has a single boundary condition causing systematic underestimation or overestimation of values. The challenge in unilateral boundary time series forecasting lies in recognizing and adjusting for these skewed segments in the data, rather than dealing with broad shifts in the overall data distribution.

### 2.2 Non-stationary time series forecasting

Non-stationary time series forecasting involves predicting future values of a time series when the statistical properties of the series, such as mean and variance, change over time. This contrasts with stationary time series forecasting, where the series is assumed to have constant statistical properties throughout. Non-stationarity poses significant challenges as it requires models to adapt to evolving trends and shifts in the data distribution.

Several recent studies have approached non-stationary time series forecasting with innovative solutions. Liu et al. (2022) introduced Non-stationary Transformers, which tackle the problem of over-stationarization that can occur when applying stationarization techniques to make a series more predictable. They propose a framework with two interdependent modules to enhance predictability while retaining the inherent non-stationary characteristics that are crucial for forecasting real-world events. Liu et al. (2023) proposed an Adaptive Normalization (SAN) that reduces non-stationarity at a local temporal slice level rather than globally across the entire series. This approach acknowledges the distribution discrepancies between different segments of the series and dynamically adjusts the model to these changes, leading to more accurate predictions. Wang et al. (2022) developed a robust forecasting framework for heavy-tailed and non-stationary time series data commonly found in finance and medical fields. They introduced an adaptive sparse Huber additive model that provides generalization bounds for both stationary and non-stationary data, circumventing the need for traditional mixing conditions and addressing shifts in data distributions over time. Lastly, Fu et al. (2022) explored a Reinforcement Learning based Model Combination (RLMC) framework that determines dynamic weights for base models in an ensemble. This method treats model selection as a sequential decision-making problem, adapting to non-stationary time series data by learning dynamic model weights and leveraging deep learning to capture hidden features from raw data.

While these methods offer advanced solutions for non-stationary time series forecasting, they differ from unilateral boundary time series forecasting in their primary focus. Unilateral boundary time series forecasting specifically addresses time series that exhibit a single boundary, either a lower or upper limit, beyond which the values are systematically underestimated or overestimated. This creates a distinct set of challenges, such as handling asymmetric error distributions and reconstructing underestimated or overestimated values, which the mentioned non-stationary forecasting methods do not directly address. Therefore, while non-stationary and unilateral boundary time series forecasting both deal with complex time series data, they require different specialized approaches to accurately predict future values.

### 2.3 Robust time series forecasting

Robust time series forecasting is concerned with the development of predictive models that maintain their accuracy and reliability in the face of anomalies, outliers, or other forms of data irregularities. These models are designed to withstand the challenges posed by noisy and unstable data, ensuring that the forecasting remains dependable even when the data inputs are imperfect or when the series experiences sudden shifts or aberrations.

Cao et al. (2023) tackle this by considering the individualized treatment effect on time series data with irregular observations and hidden confounders. They use Lipschitz regularization and neural controlled differential equations to model dynamic causal relationships in irregular samples. Yoon et al. (2022) take a probabilistic approach to robust forecasting, focusing on input perturbations and extending randomized smoothing to attain robust forecasters against adversarial perturbations. Zhang A. et al. (2023) address the robustness of Recurrent Neural Networks (RNNs) concerning input noises by minimizing the localized stochastic sensitivity, thereby enhancing the model's resilience to slight input disturbances. Zhang W. et al. (2023) propose a co-training approach for noisy time series learning, leveraging complementary information from different views to improve the robustness of the representation learning. Zeng and Li (2021) introduce a Bayesian median autoregressive model that utilizes time-varying quantile regression at the median for robust forecasting, which is inherently more resistant to outliers than mean-based methods. Wen et al. (2020) extend the RobustSTL method to handle complex patterns and multiple seasonality in time series data, greatly enhancing computational efficiency and robust decomposition capabilities.

Robust time series forecasting differs from unilateral boundary time series forecasting in its approach to handling data irregularities. While robust forecasting methods are designed to withstand various data imperfections and maintain performance in the face of such challenges, unilateral boundary time series forecasting specifically addresses the issue of systematic underestimation or overestimation within a series. This latter approach requires not only resilience to irregularities but also the ability to recognize and correct for one-sided biases that are characteristic of unilateral boundaries. Thus, while both fields aim to enhance forecasting accuracy, they do so by addressing different types of data integrity issues.

A recent work (Yang et al., 2023) addresses the challenge of time series forecasting in the presence of outliers and random noise by introducing a novel loss function (adaptive rescaled lncosh) that flexibly switches among L1, L2, and Huber losses to enhance robustness against non-standard data distributions. In contrast, our unilateral boundary time series forecasting method specifically targets datasets with systematic underestimations or overestimations, employing a dual model structure, feature reconstruction, and an asymmetric loss function to directly address these systematic biases rather than focusing on random noise and outliers. While both approaches aim to improve forecasting reliability, our method is uniquely designed to handle the challenges posed by unilateral boundary conditions in time series data, differing fundamentally in focus and technique from the robust adaptive mechanisms discussed in the referenced paper.

Another recent work, TemporalSVR (Wu et al., 2024), enhances the standard Support Vector Regression's (SVR) ability to handle time series by incorporating temporal correlations through extended kernel functions and an iterative training approach. This method addresses the limitations of traditional SVR in capturing the underlying temporal structures of correlated data, thus improving prediction efficiency. In contrast, our unilateral boundary time series forecasting method specifically addresses the issue of systematic underestimation or overestimation inherent to datasets with unilateral boundaries. Unlike the TemporalSVR, which focuses on enhancing general SVR for better temporal pattern learning across typical time series data, our method employs a dual model structure, feature reconstruction, and an asymmetric loss function tailored to correct biased data points directly linked to boundary conditions. This approach allows for targeted adjustments in forecasts, making it distinct in its focus and application compared to the generalized improvements offered by TemporalSVR.

### 2.4 Summary and comparison

The comparative analysis presented in Table 1 elucidates distinct approaches to time series forecasting, emphasizing the specific challenges each method addresses and the technological frameworks employed. Studies like Duan et al. (2023) and Fan et al. (2023) focus predominantly on distribution shifts, employing innovative methods such as dual coefficient networks and hypernetwork frameworks, respectively, to adapt dynamically to changes within data distributions. While these methods show adeptness in handling broad distributional changes, they do not specifically tackle issues related to data boundaries or systematic biases such as unilateral underestimation or overestimation. On the other hand, Kim et al. (2021) and Wang et al. (2023) introduce methods that focus on adjusting statistical properties and applying linear Koopman spaces, enhancing the robustness of forecasting against distribution shifts without directly addressing boundary-specific challenges.

**Table 1:** Comparison of time series forecasting approaches.

**Study**	**Method focus**	**Data irregularities**	**Boundary handling**	**Model adaptation**	**Methodology**
Fan et al. (2023)	Distribution shift	No	No	Yes	Dual coefficient network
Duan et al. (2023)	Distribution shift	No	No	Dynamic	Hypernetwork
Kim et al. (2021)	Statistical properties	No	No	Reversible	Instance normalization
Wang et al. (2023)	Distribution shift	No	No	Linear Koopman space	Neural networks
Cai et al. (2023)	Concept drift	Yes	No	Dynamic	Meta-dynamic network
Chen et al. (2023)	Distribution shift	No	No	Contextual	Transformers
Our work	Unilateral Boundary	Yes	Yes	Dynamic	Dual model, feature reconstruction

Further, Cai et al. (2023) deals with concept drift by utilizing a meta-dynamic network, which allows for real-time adaptability in the model's response to evolving data characteristics, highlighting an advanced approach to managing non-stationary data environments. Chen et al. (2023) enhances the adaptability of Transformers for forecasting by focusing on context-driven distribution shifts, which, although innovative, also do not cater specifically to handling unilateral boundary conditions.

In contrast, our approach uniquely addresses the challenge of unilateral boundary time series forecasting by explicitly managing both underestimated and overestimated data points through a sophisticated dual model structure and feature reconstruction technique. This method not only acknowledges but directly intervenes in the irregularities and systematic biases introduced by boundary conditions, offering a tailored solution that ensures more accurate and reliable forecasts in these specific scenarios. This direct focus on unilateral boundaries sets our work apart, providing a specialized solution where traditional and other advanced forecasting methods may not perform optimally.

## 3 Problem statement

This section precisely delineates the challenges and nuances of Unilateral Boundary Time Series Forecasting, which is the central focus of this study. It thoroughly outlines the mathematical formulation of the problem and discusses the specific scenarios where data is systematically underestimated or overestimated due to unilateral boundaries. This section serves as a foundation for the paper, as it not only defines the scope of the investigation but also justifies the necessity for the development of the novel methodologies detailed in Section 4. By establishing a clear problem statement, it links the gaps identified in the existing literature reviewed in Section 2 with the innovative solutions presented in subsequent sections, ensuring a coherent progression of the narrative throughout the paper.

In addressing the challenge of *Unilateral Boundary Time Series Forecasting*, we delve into a problem space where certain values in a time series dataset are systematically underestimated, characterized by a specific threshold. This scenario requires a methodical approach to predict future values while accounting for the skewed segments in the training dataset. The following detailed mathematical formulation and analysis outline the structure of this problem:

Consider a collection of time series data, each denoted as *X*^(*i*)^, where *i* indexes the individual time series in our dataset. Each series *X*^(*i*)^ comprises a sequence of values $x_{1}^{\left(i\right)},x_{2}^{\left(i\right)},\ldots ,x_{n}^{\left(i\right)}$ corresponding to observations at times 1, 2, …, *n*, which form the training dataset. Alongside this, we have the actual time series *Y*^(*i*)^, representing the true values that we aim to predict.

### 3.1 Model inputs and features

The forecasting model inputs consist of sequences from multiple time series, each represented as $X^{\left(i\right)}=\left\{x_{1}^{\left(i\right)},x_{2}^{\left(i\right)},...,x_{n}^{\left(i\right)}\right\}$, where *i* indexes the individual time series in our dataset, and *n* represents the number of observations in each series. These inputs include not only the historical values but also potentially derived features such as rolling averages, differences, or other transformations aimed at capturing temporal dependencies and trends relevant to the forecasting problem.

### 3.2 Model output (label)

The output of the model, or the label, is the forecasted future values of the time series, denoted as ${Ŷ}^{\left(i\right)}=\left\{x_{n+1}^{\left(i\right)},x_{n+2}^{\left(i\right)},...,x_{T}^{\left(i\right)}\right\}$ for times *n*+1 to *T*. These predictions aim to provide corrected estimates that account for the identified underestimations, aligning closely with the actual, unobserved future values of the time series.

The core of our problem involves defining and handling underestimated values within these series. We introduce a threshold parameter λ to distinctly identify these underestimated values. Specifically, a value $x_{t}^{\left(i\right)}$ in the series *X*^(*i*)^ is considered underestimated if it falls below this threshold λ. Mathematically, this can be represented as: $x_{t}^{\left(i\right)}<\lambda ,\text{where}t\in \left\{1,2,\ldots ,n\right\}$. The forecasting model, denoted as *f*(*X*^(*i*)^; Θ), where Θ signifies the model parameters, aims to map the input series *X*^(*i*)^ to the forecasted series Ŷ^(*i*)^. The model seeks to predict future values $x_{n+1}^{\left(i\right)},x_{n+2}^{\left(i\right)},\ldots ,x_{T}^{\left(i\right)}$ (for times *n*+1 to *T*) based on the training data *X*^(*i*)^, given by: Ŷ^(*i*)^ = *f*(*X*^(*i*)^; Θ).

To evaluate and optimize the model, a loss function *L*(*Y*^(*i*)^, Ŷ^(*i*)^) is employed. This function quantifies the divergence of the forecasted values Ŷ^(*i*)^ from the actual values *Y*^(*i*)^. The optimization objective is to minimize this loss function, thereby aligning the forecasted values closely with the actual ones, given by: $\underset{\Theta }{\min}\underset{i}{\sum }L\left(Y^{\left(i\right)},{Ŷ}^{\left(i\right)}\right)$.

Addressing the unilateral boundary condition in our model involves not only forecasting future values but also recognizing and adjusting for the underestimation inherent in the training data *X*^(*i*)^. This requires the model to be sensitive to the threshold λ and to adaptively adjust its predictions for values that are below this threshold.

## 4 The proposed method

This section serves as a cornerstone of our research, introducing a comprehensive framework designed to address the unique challenges of unilateral boundary time series forecasting. This section is structured into several subsections to cover various dimensions of our novel approach. It begins with an overview of our dual model structure, which processes data through bifurcated pathways to handle underestimated and accurately estimated data separately, enhancing model responsiveness and accuracy. Following this, the feature reconstruction process is detailed, explaining how we recapture the obscured dynamics of underestimated data to provide a more accurate prediction base. Additionally, the section elaborates on our innovative asymmetric loss function, the Unilateral Mean Squared Error (UMSE), which fine-tunes the model's error sensitivity to specifically address underestimation bias. Together, these subsections synthesize a robust methodology that significantly advances the predictive precision of time series forecasting under unilateral boundary conditions.

**Figure 1:**
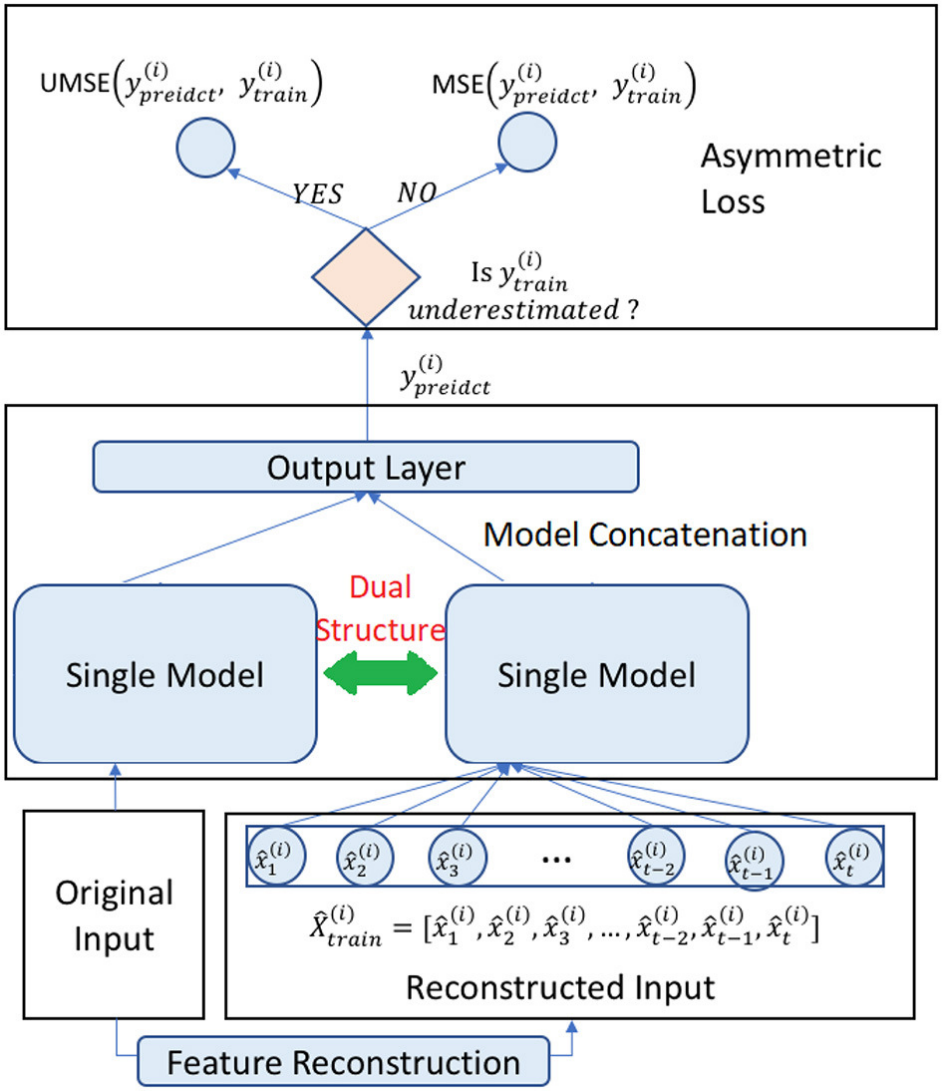
Overview of the proposed method.

We give the overview of the proposed method in Figure 1. At the outset, we present a *Dual Model Structure*, where the input data is processed through two parallel pathways within a single model framework. This bifurcated approach allows the model to treat underestimated and regularly estimated data distinctly, ensuring that each type of data is given appropriate consideration during the analysis. Following the dual structure, the process advances to *Feature Reconstruction*. In this phase, the original input data is refined to produce a reconstructed series that aims to correct for any underestimation present in the initial data. This step is crucial for restoring the integrity of the data that may have been compromised due to systematic biases, thereby preparing a more accurate foundation for the subsequent forecasting. The final component of the methodology is the implementation of an *Asymmetric Loss Function* during the training phase. Depending on whether the data has been identified as underestimated, the model selectively applies either the Unilateral Mean Squared Error (UMSE) for underestimated points or the standard Mean Squared Error (MSE) for other points. This selective application is key to tailoring the model's learning to the specific characteristics of the data, allowing it to more effectively learn from past inaccuracies.

The methodology culminates with the model leveraging the reconstructed features, informed by the dual structure analysis and refined through the asymmetric loss function, to make its final predictions. By incorporating these tailored components—dual structure processing, feature reconstruction, and asymmetric loss—the method provides a sophisticated approach to addressing the challenges of unilateral boundary forecasting. The integration of these elements ensures that the forecasting is robust, accounting for both the raw and adjusted representations of the time series data, ultimately leading to more accurate predictive outcomes.

### 4.1 Dual model structure

When grappling with datasets characterized by unilateral underestimation, we devise a novel dual model structure. Traditional forecasting methods often process input data as a singular, homogeneous entity, which, while effective in standard scenarios, falls short in accurately addressing datasets with systematic underestimation. This shortfall is primarily due to their inability to distinguish between and appropriately respond to the differences in underestimated and accurately estimated data points. The dual model structure, therefore, is proposed and conceptualized to fill this gap. It bifurcates the input dataset into two distinct streams, each tailored to either the underestimated or the non-underestimated segments of the data. This bifurcation is not merely a procedural alteration but a strategic recalibration of the model's approach to data processing. By doing so, it allows for a more nuanced and specialized analysis of each data segment, ensuring that the unique characteristics and patterns inherent in the underestimated data are given due consideration. This focused approach is crucial in environments where underestimation is a consistent feature, not an anomaly, enabling the model to learn complex patterns for forecasting that are attuned to the specific challenges posed by such data. The dual model structure, therefore, stands as a significant advancement, enhancing the accuracy and reliability of forecasts in scenarios plagued by unilateral underestimation.

#### 4.1.1 Dual LightGBM

Given an input time series for the *i*-th dataset, $\left\{x_{1}^{\left(i\right)},x_{2}^{\left(i\right)},\ldots ,x_{n}^{\left(i\right)}\right\}$, a traditional model might consider this series as a single input dimension of size *n*. In contrast, the dual model approach extends this input series into a dimension of size 2*n*, with each point being processed through two different functions, *g*_1_() and *g*_2_() (Equations 1, 2):


(1) $$g_{1}(x_{j}^{\left(i\right)})=\{\begin{array}{ll} x_{j}^{\left(i\right)} & ,\text{if }x_{j}^{\left(i\right)}\;\;\text{is underestimated}\\ NaN & ,\text{otherwise} \end{array}$$

(2) $$g_{2}(x_{j}^{\left(i\right)})=\{\begin{array}{ll} NaN & ,\text{if }x_{j}^{\left(i\right)}\;\text{is underestimated}\\ x_{j}^{\left(i\right)} & ,\text{otherwise} \end{array}$$

Here, *NaN* (Not a Number) represents an undefined or missing value in computing. Models like LightGBM (Ke et al., 2017) treat *NaN* values as missing data, effectively ignoring them during decision splits. In implementing this structure with LightGBM, the extended input series becomes $\left\{g_{1}\left(x_{1}^{\left(i\right)}\right),g_{1}\left(x_{2}^{\left(i\right)}\right),\ldots ,g_{1}\left(x_{n}^{\left(i\right)}\right),g_{2}\left(x_{1}^{\left(i\right)}\right),g_{2}\left(x_{2}^{\left(i\right)}\right),\ldots ,g_{2}\left(x_{n}^{\left(i\right)}\right)\right\}$. The first half of this series retains the underestimated data while setting non-underestimated data to *NaN*. Conversely, the second half keeps the non-underestimated data, assigning NaN to the underestimated ones. This bifurcation allows the LightGBM model to process and weigh these two data streams separately.

The efficacy of the Dual LightGBM model in addressing underestimation in unilateral time series forecasting is rooted in its approach to data segregation and processing. By dividing the input data into two distinct streams, the model adeptly isolates underestimated data points from their non-underestimated counterparts. This bifurcation is crucial, as it allows each subset of data to be analyzed independently, ensuring that the unique characteristics of underestimated data are not overshadowed by the general trends of the complete dataset. The Dual LightGBM model leverages this separation to apply specialized treatment to the underestimated data, enhancing the model's sensitivity to subtle nuances that standard approaches might overlook. This heightened sensitivity is key in accurately predicting values that are prone to underestimation, as it enables the model to compensate for biases inherent in the data.

#### 4.1.2 Dual GRU

The Gated Recurrent Unit (GRU) (Cho et al., 2014) has established itself as a pivotal component in the realm of recurrent neural networks, particularly for time series forecasting. Its standard mathematical formulation is well-known for effectively handling sequential data. However, in the context of time series data characterized by unilateral boundaries like underestimation, the standard GRU structure requires modifications to address the unique challenges posed by such data. This subsection explores the adaptations to the GRU model to better handle underestimation issues in time series forecasting.

The standard GRU cell's mathematical operations are governed by the following equations (Equation 3):


(3) $$\begin{array}{l} z_{t}=\sigma (W_{z}x_{t}+U_{z}h_{t-1}+b_{z})\\ r_{t}=\sigma (W_{r}x_{t}+U_{r}h_{t-1}+b_{r})\\ {\tilde{h}}_{t}=\tanh(Wx_{t}+U(r_{t}⊙h_{t-1})+b)\\ h_{t}=(1-z_{t})h_{t-1}+z_{t}{\tilde{h}}_{t} \end{array}$$

In these equations, *x*_*t*_ represents the new input for the GRU cell at time *t*, and *h*_*t*−1_ is the hidden state from the previous time step. The GRU cell uses gates (update gate *z*_*t*_ and reset gate *r*_*t*_) to regulate the flow of information through the unit, effectively capturing dependencies over different time scales. We propose a variant of GRUs that is specialized to deal with underestimation in uniliteral boundary time series forecasting.

A straightforward method to tackle underestimation in time series data is to modify the GRU's gating mechanism. One such approach, involves setting the update gate *z*_*t*_ to zero whenever the input value *x*_*t*_ is underestimated. This prevents the underestimated value from influencing the time series embedding process within the GRU. However, this approach has a significant downside: it results in the loss of information from underestimated values, which could be vital for accurate forecasting. To overcome the limitations of the GRU-close approach, a modified method duplicates certain parameters within the GRU. This setup involves creating two sets of GRU parameters: one set for normal values and another for underestimated values in the time series. The duplicated parameters include *W*_*z*_, *W*_*r*_, *W, U*_*z*_, *U*_*r*_, *U, b*_*z*_, *b*_*r*_, and *b*. The idea here is to allow the GRU to process normal and underestimated values separately, using distinct parameter sets for each. We propose to further selectively duplicate parameters within the GRU. Only the parameters that deal directly with the input values (*W*_*z*_, *W*_*r*_, *W, b*_*z*_, *b*_*r*_, *b*) are duplicated. This decision is based on the understanding that normal and underestimated values exhibit different distributions and, hence, require distinct processing. Conversely, parameters associated with the previous time step's hidden state (*U*_*z*_, *U*_*r*_, *U*) remain unchanged. The rationale behind this is that the time series embedding, which captures the temporal dynamics of the series, should be consistent, regardless of whether the current input value is normal or underestimated.

Traditionally, GRU models, with their powerful sequential data processing capabilities, have been hampered by their inability to differentiate between normal and underestimated data points. The dual GRU model overcomes this limitation by introducing a bifurcated processing pathway – one that treats underestimated and normal data separately, thereby tailoring the model's response to the unique characteristics of each data type. In unilateral time series, underestimated data points are not merely outliers or random noise; they represent a consistent and systematic deviation from the true values. Standard models tend to absorb these deviations, leading to a compounding of errors in forecasts. The dual GRU, particularly in its GRU-2w variant, addresses this by selectively duplicating parameters related to input processing. This targeted approach ensures that the model does not overgeneralize from the skewed data, a critical factor in preventing the perpetuation of underestimation biases. By providing separate pathways for underestimated data, the model ensures that these points do not disproportionately influence the overall forecasting. This leads to more reliable and accurate predictions.

### 4.2 Feature reconstruction

Feature reconstruction in unilateral time series forecasting is a critical procedure aimed at reconstructing the actual values from a partially underestimated input series. This process is particularly important in addressing the challenges posed by systematic underestimation in time series data. In this subsection, we will describee the detailed steps involved in feature reconstruction, its application in LightGBM and GRU models, and discuss how this approach contributes to more accurate forecasting.

The process of feature reconstruction involves several key steps, each designed to enhance the model's ability to predict accurate values by compensating for the underestimation inherent in the input data.

1. Model training with dual structure: initially, a model (referred to as model_A) is trained using the dual model Structure. This model is specifically designed to handle underestimated and normal values separately, as described in the dual model subsections for LightGBM and GRU.
2. Prediction of reconstructed input: utilizing the original input series and the trained model_A, the reconstructed input series is predicted. This step is crucial as it aims to estimate what the actual values of the input series might have been, had there been no underestimation.
3. Training of Secondary model: subsequently, a second model (model_B) is trained using the reconstructed input series along with the original input data.
4. Testing phase prediction: in the testing phase, the first step involves using model_A to predict the reconstructed testing input from the original testing input. This reconstructed testing input, along with the original testing input, is then used to make the final prediction of the testing output using model_B.

#### 4.2.1 Model concatenation in LightGBM

In the context of LightGBM, feature reconstruction involves a concatenation process. Given an original input series $X=\left\{x_{1}^{\left(i\right)},x_{2}^{\left(i\right)},\ldots ,x_{n}^{\left(i\right)}\right\}$ and a reconstructed input series $\hat{X}=\left\{{\hat{x}}_{1}^{\left(i\right)},{\hat{x}}_{2}^{\left(i\right)},\ldots ,{\hat{x}}_{n}^{\left(i\right)}\right\}$, the concatenated input series for Light GBM is represented as Equation 4:


(4) $$\begin{array}{r} {\hat{x}}_{1}^{\left(i\right)},{\hat{x}}_{2}^{\left(i\right)},\ldots ,{\hat{x}}_{n}^{\left(i\right)},g_{1}\left(x_{1}^{\left(i\right)}\right),g_{1}\left(x_{2}^{\left(i\right)}\right),\ldots ,g_{1}\left(x_{n}^{\left(i\right)}\right),g_{2}\left(x_{1}^{\left(i\right)}\right),g_{2}\left(x_{2}^{\left(i\right)}\right),\\ \ldots ,g_{2}\left(x_{n}^{\left(i\right)}\right). \end{array}$$

Here, the functions *g*_1_() and *g*_2_(), as defined in the dual model structure, are applied to the original series, and the resulting series is concatenated with the reconstructed series $\hat{X}$. The resulting input dimension for the Light GBM model is 3*n*, incorporating the original, the dual processed, and the reconstructed series.

#### 4.2.2 Model concatenation in GRU

For the GRU model, the concatenation process involves feeding the original input series *X* to the dual GRU model and the reconstructed input series $\hat{X}$ to a standard GRU model. The outputs of these two GRU models are then concatenated and passed through a dense layer for final processing. This approach allows the model to leverage the strengths of both the dual GRU (handling underestimated and normal values) and the standard GRU (processing the reconstructed series), thereby enhancing the overall predictive capability.

Feature reconstruction addresses a critical limitation in time series forecasting models dealing with unilateral underestimation. By reconstructing the actual values from underestimated data, this approach provides a more accurate representation of the true nature of the input series. This, in turn, allows the forecasting models (both LightGBM and GRU) to make predictions based on a more realistic and comprehensive understanding of the data. The concatenation of the original, dual processed, and reconstructed series in both LightGBM and GRU models ensures that the forecasting is informed by a holistic view of the data. It combines the insights gained from the original series, the nuanced understanding from the dual-processed series, and the corrected perspective from the reconstructed series.

### 4.3 Asymmetric loss function

We propose the asymmetric loss function, specifically the *Unilateral Mean Squared Error* (UMSE), to addresse the challenge of handling underestimated values in time series forecasting. The design and application of this loss function are crucial in mitigating underestimation issues, leading to more accurate and reliable forecasting outcomes. In this section, we will elaborate the rationale behind the design of UMSE, its mathematical formulation, and how it effectively addresses the issue of underestimation in time series data.

The Mean Squared Error (MSE), a traditional loss function widely used in regression problems, is defined as follows Equation 5:


(5) $$\begin{array}{l} MSE\left(Ŷ,Y\right)=\frac{1}{n}\overset{n}{\underset{t=1}{\sum }}{\left({Ŷ}_{t}-Y_{t}\right)}^{2} \end{array}$$

However, this standard MSE does not specifically cater to the nuances of time series data with unilateral boundaries. In such cases, a segment of the training data may be systematically underestimated. To address this, we propose the Unilateral Mean Squared Error (UMSE) as an asymmetric loss function, given by Equation 6:


(6) $$\begin{array}{l} UMSE\left(Ŷ,Y\right)=\frac{1}{n}\overset{n}{\underset{t=1}{\sum }}\left[1-H\left({Ŷ}_{t}-Y_{t}\right)\right]\cdot {\left({Ŷ}_{t}-Y_{t}\right)}^{2}, \end{array}$$

where *H*(*x*) represents the Heaviside step function, given by Equation 7:


(7) $$\begin{array}{l} H\left(x\right)=\frac{d}{dx}\left[\max\left(x,0\right)\right],\text{for }x\neq 0. \end{array}$$

The UMSE loss function is designed to differentially penalize errors based on whether they stem from underestimated values. This is achieved through the incorporation of the Heaviside step function, which serves as a switch to modulate the contribution of each term in the loss function based on the relationship between the predicted value Ŷ_*t*_ and the training value *Y*_*t*_.

- When *Y*_*i*_>Ŷ_*i*_, implying that the forecasted value is smaller than the underestimated training value, UMSE aligns with the traditional MSE. This is logical because, in such scenarios, the actual value is assumed to be greater than the underestimated value. Hence, the error should be treated as it would be in standard regression problems, focusing on minimizing the squared error.
- Conversely, when *Y*_*i*_ < Ŷ_*i*_, indicating that the forecasted value exceeds the underestimated value, UMSE is set to zero. This design choice is particularly insightful. It stems from the uncertainty regarding the relationship between the predicted value and the actual value. If the predicted value surpasses the underestimated value, it is unclear without additional information whether the prediction is overestimating, accurate, or still underestimating the true value. Therefore, penalizing such predictions could potentially lead to further underestimation, defeating the purpose of the forecasting model.

In time series forecasting, especially with unilateral boundaries, accurately predicting future values hinges on the model's ability to learn from and adjust for systematic underestimations in the training data. The UMSE function directly addresses this challenge by effectively “ignoring” instances where the model predicts values higher than the underestimated training values. This approach encourages the model to *err on the side of overestimation*, countering the inherent bias toward underestimation in the data.

Moreover, the asymmetric nature of UMSE ensures that the model is not excessively penalized for overestimations, which is crucial in contexts where underestimation poses a greater risk or cost. This is particularly relevant in scenarios like inventory management or demand forecasting, where underestimation can lead to more severe consequences than overestimation.

### 4.4 Algorithm

The Algorithm 1 detailed above provides a structured approach to forecast time series data with considerations for unilateral boundary conditions, specifically focusing on systematic underestimations within the dataset. Initially, the algorithm initializes the model parameters and processes each series within the dataset to identify underestimated data points based on a predefined threshold, λ. It then extracts and reconstructs features to correct any biases induced by these underestimations. The dataset is split into training and validation sets, and the model iteratively learns by adjusting its parameters through a training loop where each batch of the reconstructed and original data is used to compute the forecast and minimize the loss using either Unilateral Mean Squared Error (UMSE) or Mean Squared Error (MSE), depending on the data's characteristics. Finally, the algorithm applies the trained model parameters to forecast future values for each series using both the original and reconstructed features, ensuring that the forecasts are robust, accurate, and reflective of the corrected data representations. This approach is not only comprehensive but also enhances the forecasting accuracy by effectively addressing the challenges of unilateral boundary conditions in time series data.

**Algorithm 1:**
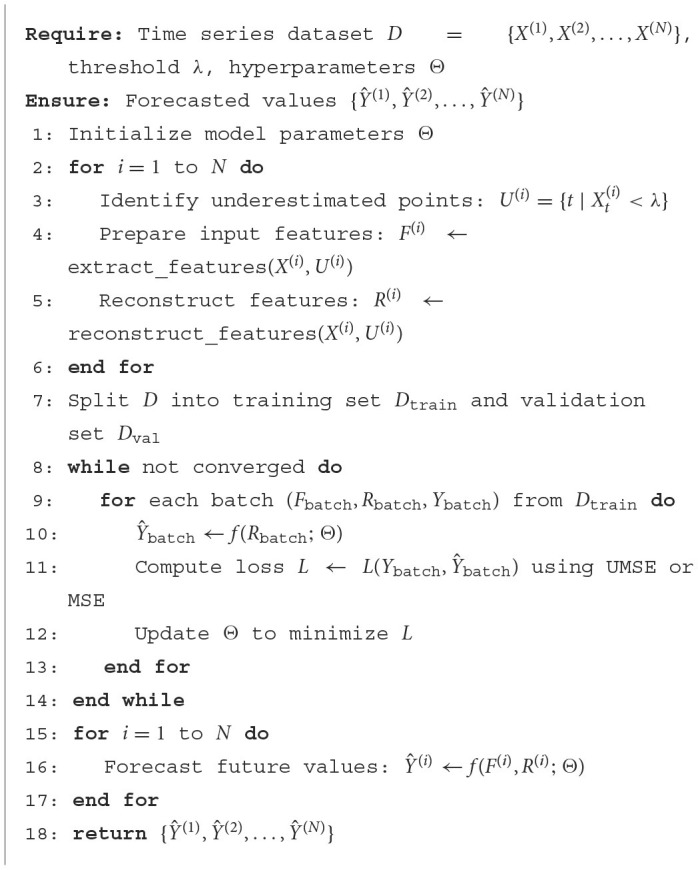
Unilateral boundary time series forecasting.

### 4.5 Computational cost and uncertainty

#### 4.5.1 Computational cost

The computational efficiency of our proposed method, which integrates a Dual Model Structure, Feature Reconstruction, and an Asymmetric Loss Function, is designed to optimize both accuracy and performance for unilateral boundary time series forecasting. Here is a detailed analysis of the computational aspects:

1. Dual model structure: this approach processes data through two parallel pathways, effectively doubling the input dimension but not necessarily doubling the computation time. Modern computing frameworks efficiently handle such parallel computations, particularly on hardware optimized for matrix operations, such as GPUs. The separation into two streams allows for targeted processing, which can be executed concurrently, thereby minimizing the increase in computational load. Moreover, this bifurcation ensures that each data type–underestimated and regularly estimated–is processed by the model that's best fit for its characteristics, reducing the number of iterations needed for the model to converge.
2. Feature reconstruction: although reconstructing features from underestimated data introduces additional steps in the forecasting process, the computational cost is mitigated by the use of sophisticated vectorized operations that are well-supported by modern machine learning libraries. This phase involves recalculating inputs which, while computationally demanding, is limited to once per training iteration. The reconstructed features enhance model accuracy, which can reduce the number of epochs required to achieve optimal results, thus indirectly lowering the overall computational expense.
3. Asymmetric loss function: the implementation of the Unilateral Mean Squared Error (UMSE) for underestimated points and standard Mean Squared Error (MSE) for other points requires conditional operations within the loss calculation. However, these operations are simple comparisons and do not significantly affect the computational complexity. The selective application of loss functions allows for focused learning, where the model spends more computational resources on difficult, underestimated cases rather than treating all data uniformly. This targeted learning approach can lead to faster convergence, thus reducing the number of gradient updates required.

Overall, the computational cost of our proposed method is efficiently managed through the use of various data processing techniques and optimized loss function calculations. The dual structure of the model leverages parallel processing capabilities, feature reconstruction is streamlined through vector operations, and the asymmetric loss function focuses computational efforts where they are most needed, leading to a cost-effective solution. This makes our approach not only innovative but also practical, balancing computational demands with forecasting performance, thus providing a robust and efficient tool for dealing with unilateral boundary challenges in time series data.

#### 4.5.2 Uncertainty

In addressing the uncertainty associated with the proposed method for Unilateral Boundary Time Series Forecasting, it is essential to emphasize the sophisticated mechanisms we have implemented to ensure robust and accurate predictions, thereby reducing uncertainty to a minimum. The integration of a dual model structure, feature reconstruction, and an asymmetric loss function specifically tailors the approach to the unique challenges posed by unilateral boundary conditions, effectively mitigating risks of model error and mispredictions.

The dual model structure allows our method to process underestimated and regularly estimated data separately, enhancing the precision of data treatment and minimizing errors caused by indiscriminate processing of divergent data types. This bifurcation ensures that each segment of data receives appropriate analysis, reducing the likelihood of error propagation throughout the forecasting process.

Feature reconstruction further minimizes uncertainty by correcting any biases introduced by underestimation in the input data. By reconstructing a more accurate series from the compromised data, this step not only restores the integrity of the information but also improves the foundation upon which forecasts are made. This process is crucial for ensuring that the predictions are not only based on accurate data but also reflect a deeper understanding of the underlying dynamics, which might otherwise be obscured by systematic biases.

Lastly, the implementation of the Unilateral Mean Squared Error (UMSE) in the training phase introduces a tailored approach to handling losses, specifically focusing on minimizing the impact of underestimated data points. By differentially penalizing errors based on their nature—whether they arise from underestimations or not—UMSE helps in refining the model's predictions. This loss function reduces the predictive uncertainty significantly by encouraging the model to compensate for potential underestimations, thus aligning forecasted outputs more closely with the true values.

Through these methodological innovations, our approach substantially lowers the inherent uncertainty typical of unilateral boundary forecasting. The method's architecture, which specifically addresses the complications introduced by boundary conditions, ensures that the forecasting model is not only robust but also remarkably precise in its output, leading to enhanced reliability and accuracy in its predictions. This comprehensive strategy effectively minimizes the uncertainty that might otherwise be prevalent in less sophisticated forecasting methodologies.

## 5 Experiments

This section rigorously tests the efficacy of our proposed method for unilateral boundary time series forecasting through a series of detailed experimental setups and evaluations. It is divided into several subsections that outline the experimental design, describe the datasets used, detail the underestimation simulation methods, and discuss the evaluation metrics employed to assess model performance. Results are then meticulously analyzed to demonstrate the superiority of our approach over existing models. The section not only provides empirical evidence supporting the theoretical advancements discussed in earlier sections but also bridges the conceptual framework with practical applicability. By comparing our results with baseline models, this section reinforces the relevance and innovation of our method, setting the stage for the concluding remarks where future research directions are proposed based on the experimental findings.

### 5.1 Evaluation settings

#### 5.1.1 Datasets

We select three distinct time series datasets, each offering unique insights into different domains: air quality measurement and urban transportation demand. These datasets are integral to our experiments, providing a diverse and challenging set of scenarios for testing our proposed unilateral boundary time series forecasting models.

##### 5.1.1.1 Beijing and London PM2.5 air quality datasets

The first dataset focuses on air quality, specifically on PM2.5 levels, in two major cities: Beijing and London. This dataset^1^ was a key component of the KDD Cup 2018 forecasting competition^2^, renowned for its comprehensive and detailed air quality records. It encompasses long hourly time series data from 59 monitoring stations, with 35 stations located in Beijing and 24 in London, spanning from January 1, 2017, to March 31, 2018. The PM2.5 dataset is particularly valuable for its intricate detailing of air quality levels, offering a granular view of environmental conditions over time. The data is meticulously categorized by city, station name, and air quality measurement, resulting in a total of 270 distinct hourly time series. This rich dataset not only provides a basis for examining air quality trends but also serves as a testing ground for our forecasting models, particularly in understanding and predicting environmental and urban air quality dynamics.

##### 5.1.1.2 Singapore taxi demand dataset

The third dataset we utilize captures the vibrant and dynamic urban transportation landscape of Singapore through the lens of taxi demand. This dataset (Kang et al., 2013) is sourced from GPS logs of over 15,000 taxicabs in Singapore, collected over an entire week. The sheer volume of data, encompassing 123,573,303 GPS points, provides a comprehensive snapshot of urban mobility. Each GPS point in this dataset includes longitude, latitude, time, and the occupancy status (occupied or unoccupied) of the taxi. The temporal granularity of the data is noteworthy, with intervals between consecutive GPS points varying according to different taxicabs and times of the day. The most common intervals are 1, 3, and 5 s. To contextualize this data spatially, we discretized Singapore's geo-spatial area into 500 m *times* 500 m grids, enabling us to construct time series data for both occupied and vacant taxis. This approach allows us to delve into the intricacies of urban taxi demand, offering insights into patterns of mobility and the factors influencing taxi occupancy rates.

#### 5.1.2 Underestimation simulation

We propose Random Perturbation Sampling to simulate underestimation in time series data. This method is particularly effective in replicating real-world situations where a value in the time series data is underestimated due to random factors. An example of such a scenario is observed in the functioning of low-cost soil water sensors, which are known to often either underestimate or overestimate soil water content without any specific reason (Ganjegunte et al., 2012). The proposed random perturbation sampling method is structured as follows:

1. Setting underestimation probability: we start by defining an underestimation probability, denoted as α. This probability determines how frequently values in the time series will be randomly underestimated. In our experiments, we varied α to different values, such as 0.25, 0.50, and 0.75, to simulate varying levels of underestimation frequency.
2. Choosing distribution functions: different distribution functions are used to simulate the underestimation. For instance, we employed Normal, Uniform, and Half-Normal distributions. These distribution choices reflect the diverse ways in which real-world underestimation can occur, ranging from systematic biases (as in the Normal distribution) to completely random fluctuations (as in the Uniform and Half-Normal distributions).
3. Applying the underestimation procedure: for each value in the original time series data $\tilde{ts}$, a random number between 0 and 1 is generated. If this number is less than the predefined probability α, the corresponding time series value is considered for underestimation. The underestimation is applied by taking the original value ${\tilde{ts}}_{i}$, reducing it by half, and then applying the chosen distribution function to this reduced value. This results in a new value *ts*_*i*_, which is used in the underestimated time series.
4. Creating the underestimated time series: if the random number is greater than or equal to α, the original value from the time series ${\tilde{ts}}_{i}$ is retained in the new time series *ts*. This step ensures that underestimation occurs randomly, as per the specified probability, and not to every data point in the series.

By varying the distributions and the underestimation probability α, for each dataset, we can generate different uniliteral boundary time series datasets, as exhibited in Table 2. This method thus offers a realistic simulation of random underestimation phenomena observed in real-world scenarios. Such kind of simulation allows us to test the robustness and adaptability of our forecasting models under diverse underestimation conditions. The insights gained from these experiments are crucial in understanding how well our models can perform in real-world situations where underestimation occurs without a specific, identifiable cause.

**Table 2:** Datasets generated by random perturbation sampling.

**Data name**	**Underestimation ratio (α)**	**Distribution**
prob25	25%	Normal (μ = 0.5)
prob50	50%	Normal (μ = 0.5)
prob75	75%	Normal (μ = 0.5)
prob50_uniform	50%	Uniform (μ = 0.5)
prob50_half	50%	Half-Normal (μ = 1)

#### 5.1.3 Evaluation metrics

In the evaluation of our forecasting models, two metrics were employed: the Root Mean Squared Error (RMSE) (Makridakis et al., 1982) and the Mean Absolute Error (MAE) (Armstrong, 2001). These metrics are crucial in assessing the accuracy and reliability of our models in predicting time series data, especially under conditions of underestimation. RMSE is a widely used measure in statistical and forecasting models, valued for its ability to quantify the magnitude of prediction errors. The formula for RMSE is given by Equation 8:


(8) $$\begin{array}{l} RMSE\left(y,ŷ\right)=\sqrt{\frac{1}{n}\overset{n}{\underset{i=1}{\sum }}{\left(y_{i}-{ŷ}_{i}\right)}^{2}}. \end{array}$$

Here, *y*_*i*_ represents the actual value and ŷ_*i*_ is the predicted value. RMSE calculates the square root of the average squared differences between these values over *n* data points. One of the primary reasons for preferring RMSE over the Mean Squared Error (MSE) is that it retains the same scale as the data, making the interpretation more intuitive. Historically, RMSE (and by extension, MSE) has been popular due to its theoretical relevance in statistical modeling.

MAE is another fundamental metric used in evaluating the performance of forecasting models. It is defined as Equation 9:


(9) $$\begin{array}{l} MAE\left(y,ŷ\right)=\frac{1}{n}\overset{n}{\underset{i=1}{\sum }}|y_{i}-{ŷ}_{i}|. \end{array}$$

This metric calculates the average of the absolute differences between predicted values and actual values. Unlike RMSE, MAE provides a direct average of error magnitudes and is not overly sensitive to outliers. This characteristic of MAE has led some researchers to advocate for its use, especially in scenarios where outliers significantly impact the model's performance.

Both RMSE and MAE offer distinct perspectives on the accuracy of forecasting models. While RMSE is more sensitive to larger errors (due to squaring the differences), giving an indication of the severity of errors, MAE offers a straightforward average of error magnitudes. This combination of metrics provides a comprehensive understanding of model performance, highlighting both the average accuracy and the impact of large deviations in predictions. In our experiments, the use of RMSE and MAE allows for a balanced evaluation of the forecasting models' ability to handle underestimation in time series data. For each dataset, we repeat the underestimation simulation 20 times over five random perturbation settings listed in Table 2, and report the average scores of MAE and RMSE.

#### 5.1.4 Competing methods

We compare the proposed methods with several established approaches, albeit designed for different purposes, to demonstrate their efficacy in handling underestimation issues. While no models are known to specifically address unilateral boundary time series forecasting, some research works can be adapted for this purpose.

##### 5.1.4.1 Deep censored learning

Originally proposed by Wu et al. (2018), Deep Censored Learning (DCL) was designed to enable deep learning models to learn from historically underestimated bidding prices. The primary distinction between DCL's target problem and unilateral boundary time series forecasting lies in the nature of underestimation. In the bidding context, underestimation occurs only in the observed bidding price (*y*_*train*_), with input features remaining unaffected. Conversely, in unilateral boundary time series forecasting, both the targeted time series value (*y*_*train*_) and input features (*X*_*train*_) can be underestimated. In our research, we introduced an asymmetric loss function (UMSE) to specifically address the underestimated training output (*y*_*train*_). This enabled us to evaluate the effectiveness of DCL against our UMSE, even without incorporating the dual model structure and feature reconstruction.

##### 5.1.4.2 GRU-D

Developed by Che et al. (2018), GRU-D is an approach tailored for multivariate time series with missing values. While GRU-D focuses on missing values in training and testing data, our work concentrates on underestimated values in these datasets. Our proposed Dual GRU model was specifically designed to address underestimated values in input data (*X*_*train*_). To assess the applicability of GRU-D in handling underestimation, we conducted comparative experiments. These experiments revealed that while GRU-D could be adapted for underestimated input data, our Dual GRU showed superior performance.

##### 5.1.4.3 LSTNet

Another method we compared our approach with is the Long- and Short-term Time-series network (LSTNet) (Lai et al., 2018). LSTNet combines Convolution Neural Network (CNN) and Recurrent Neural Network (RNN) architectures to extract short-term local dependency patterns and discover long-term trends in time series. In addition, it employs a traditional autoregressive model to address the scale insensitivity issue common in neural network models. LSTNet has demonstrated significant improvements over several state-of-the-art baseline methods in real-world data evaluations.

By comparing our methods with these established approaches, we aimed to showcase the robustness and adaptability of our models in unilateral boundary time series forecasting. Our findings from these comparisons affirm that our methodologies, particularly UMSE and Dual GRU model, are not only suitable for handling underestimation in time series data but also outperform existing models that were adapted for this purpose.

### 5.2 Experimental results

In our experiments, we sought to address several critical evaluation questions. These questions were carefully crafted to dissect the effectiveness of our proposed methods and to compare them with existing techniques. The objective was to validate the performance of our models under various conditions and to understand the influencing their effectiveness.

1. Effectiveness of asymmetric loss function: a primary question was whether incorporating an asymmetric loss function improves forecasting accuracy. Specifically, we wanted to assess if our Unilateral Mean Squared Error (UMSE) approach yields better results than Deep Censored Learning (DCL). This evaluation aimed to ascertain the impact of our tailored loss function on handling underestimation in time series data.
2. Dual model structure utility: another crucial question was the effectiveness of the dual model structure. We examined whether our dual GRU model outperforms GRU-D in unilateral boundary time series forecasting. This comparison was essential to validate whether the dual model approach, designed to process underestimated and accurately estimated values separately, offers a significant advantage over traditional methods.
3. Benefits of feature reconstruction: we also explored the effectiveness of feature reconstruction in enhancing the forecasting accuracy. This technique involves reconstructing the actual values from underestimated data, and its efficacy was assessed in improving the model's predictive performance.
4. Comparative performance against LSTNet and DCL: to establish the relative performance of our proposed model, we compared it with LSTNet. This comparison aimed to determine if our model offers any substantial improvements over established methods in handling time series data.

Through these evaluation questions, our experiments were structured to not only validate the effectiveness of our methodologies but also to provide deeper insights into their functioning and advantages over existing methods in unilateral boundary time series forecasting.

#### 5.2.1 Main results

**Figure 2:**
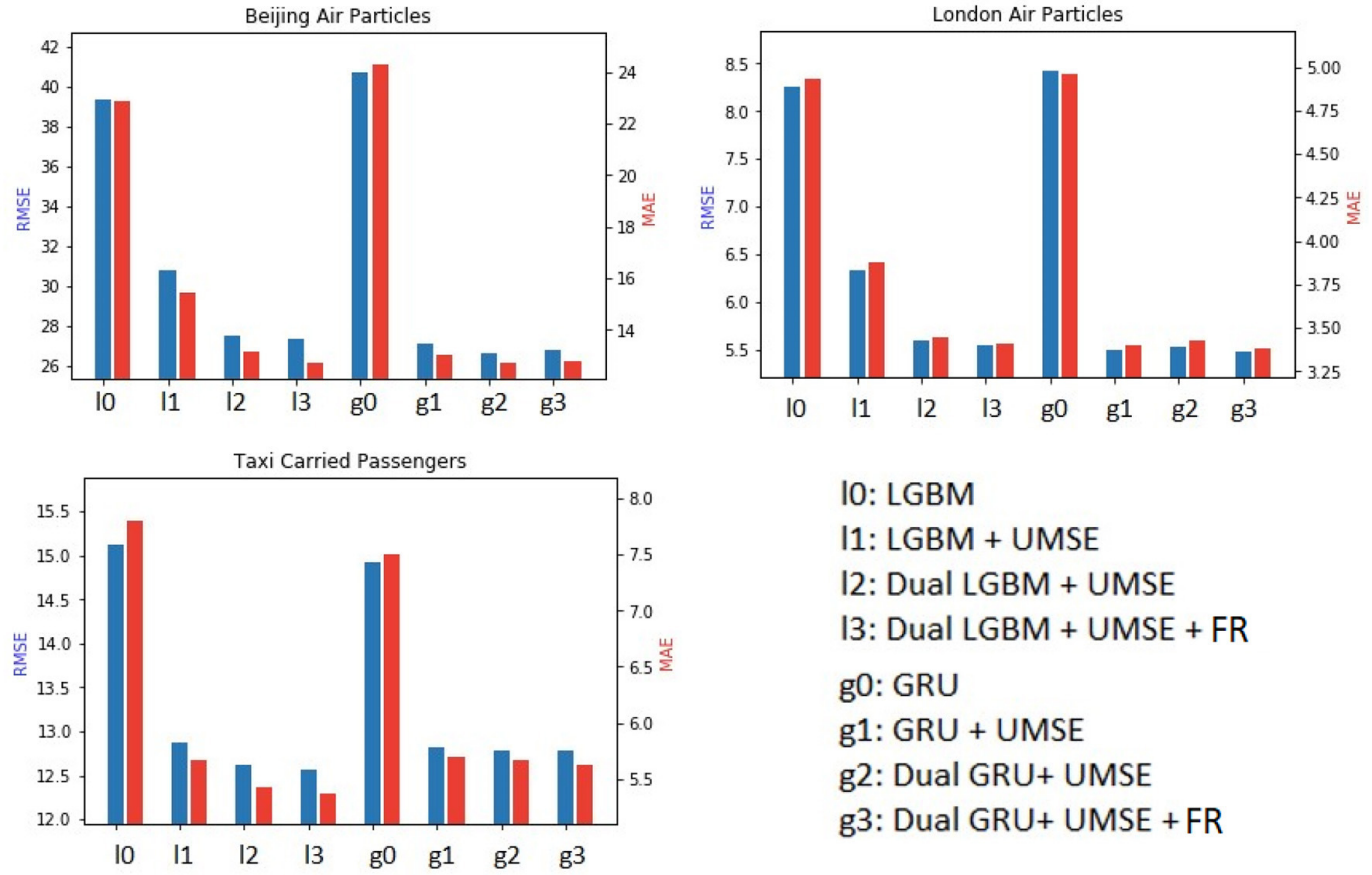
Main results with ablation study in terms of MAE and RMSE for the three datasets.

The experimental results depicted in Figure 2 point to significant insights in the task of unilateral boundary time series forecasting, especially when using a dual model approach with feature reconstruction (FR) and Unilateral Mean Squared Error (UMSE).

In both Beijing Air Particles and London Air Particles datasets, the traditional LightGBM (l0) method provides a baseline for comparison. The addition of UMSE (l1) improves upon this, suggesting that UMSE's asymmetry is effective in correcting unilateral underestimation. The Dual LightGBM with UMSE (l2) further enhances forecasting accuracy, affirming the hypothesis that treating underestimated and accurately estimated data points differently is beneficial. Incorporating feature reconstruction (l3) achieves the best results, implying that reconstructing underestimated features allows the model to adjust its predictions more precisely. The GRU model exhibits a similar trend. The base GRU model (g0) is outperformed by the GRU with UMSE (g1), again highlighting the importance of addressing unilateral underestimation directly within the loss function. The Dual GRU with UMSE (g2) surpasses the single-stream GRU with UMSE, reinforcing the dual model's efficacy. Lastly, the addition of feature reconstruction to the Dual GRU with UMSE (g3) yields the most accurate forecasts, underscoring the critical role of feature reconstruction in forecasting when systematic underestimation is present. Notably, for the Taxi Carried Passengers dataset, the improvements are particularly striking with the addition of UMSE and feature reconstruction, showcasing their crucial roles in forecasting accuracy.

Furthermore, we can have the following findings based on the ablation study.

- The dual model structure's bifurcation of the input dataset into underestimated and non-underestimated streams enables a tailored approach to each segment, enhancing model learning and forecasting precision. This is a significant advancement for environments where underestimation is a consistent feature and not an anomaly.
- Feature reconstruction's role in this context cannot be overstated. By reconstructing actual values from a partially underestimated input series, it allows the model to make more accurate predictions by “filling in the gaps” in the data that traditional methods may overlook. This is especially evident in the results for the Taxi Carried Passengers dataset, where feature reconstruction in both LightGBM and GRU models (l3 and g3) led to the most notable improvements in forecasting accuracy.
- The use of UMSE is a pivotal component in these experiments. It provides a targeted approach to handling underestimation by introducing an asymmetric loss function that “ignores” overpredictions relative to the underestimated data points. This strategic choice encourages the model to err toward overestimation, which is particularly beneficial in scenarios where the cost of underestimation is greater than overestimation. The asymmetric property of UMSE ensures that the model is not excessively penalized for overestimations, which is crucial for maintaining a balance between sensitivity to underestimation and the avoidance of excessive penalty for overestimation.

#### 5.2.2 Effectiveness of asymmetric loss function

Tables 3, 4 offer a comprehensive evaluation of the asymmetric loss function, specifically the UMSE, in the context of unilateral boundary time series forecasting. The results reveal insights into the performance of UMSE when applied to different forecasting models and datasets.

- For both MAE and RMSE across all datasets, the integration of UMSE with LightGBM and GRU models results in the lowest error metrics, denoted by bold figures that have passed statistical significance testing via t-tests against both the baseline model and the Deep Censored Learning (DCL) model. This indicates that UMSE provides a substantial improvement over the baseline and even over the advanced DCL method, with a p-value of 0.05 indicating a high level of statistical significance.
- In the case of LightGBM, the addition of UMSE results in a decrease in both MAE and RMSE across datasets. These results are not only statistically significant but also indicate a meaningful improvement in forecasting accuracy, emphasizing the effectiveness of UMSE in correcting the model's inherent underestimation bias. The GRU model's results echo this trend, where the implementation of UMSE demonstrates even more pronounced improvements. The statistical significance confirms that UMSE is not only beneficial but also a statistically reliable enhancement over traditional loss functions and DCL in this context. The results strongly suggest that UMSE's asymmetric design, which focuses on penalizing the underestimation of unilateral boundaries more than overestimation, aligns well with the inherent distribution of the datasets in question.
- Moreover, these results underscore the adaptability of UMSE across different machine learning models. Both LightGBM, which is gradient boosting-based, and GRU, a type of recurrent neural network, benefit from the integration of UMSE, demonstrating the loss function's versatility and robustness. This cross-model efficacy is particularly notable as it suggests that UMSE can be a broadly applicable tool in the machine learning toolkit for time series forecasting, regardless of the underlying algorithm.

**Table 3:** Results in MAE on evaluating unilateral MSE loss.

**Model**	**Beijing**	**London**	**Taxi**
LightGBM	22.861	4.934	7.796
LightGBM + DCL	15.473	4.226	5.678
LightGBM + UMSE	15.431^†‡^	3.875^†‡^	5.663^†‡^
GRU	24.444	5.004	7.488
GRU + DCL	14.595	3.805	5.739
GRU + UMSE	13.018^†‡^	3.430^†‡^	5.683^†‡^

^†^Indicates passing *t*-test comparing with baseline model (*p*-value = 0.05).

^‡^Indicates passing t-test comparing with DCL (*p*-value = 0.05).

**Table 4:** Results in RMSE on evaluating unilateral MSE loss.

**Model**	**Beijing**	**London**	**Taxi**
LightGBM	39.380	8.251	15.128
LightGBM + DCL	30.996	6.434	12.877
LightGBM + UMSE	30.769^†‡^	6.326^†‡^	12.867^†‡^
GRU	40.860	8.512	14.913
GRU + DCL	28.007	5.958	12.846
GRU + UMSE	27.216^†‡^	5.538^†‡^	12.817^†^

^†^Indicates passing *t*-test comparing with baseline model (*p*-value = 0.05).

^‡^Indicates passing t-test comparing with DCL (*p*-value = 0.05).

#### 5.2.3 Dual model structure utility

The evaluation of the dual model structure, as evidenced by Tables 5, 6, demonstrates a clear and consistent advantage over traditional single-stream models. By integrating a dual structure with UMSE, the forecasting accuracy is notably enhanced across various datasets, indicated by both MAE and RMSE improvements. The dual structure's utility is evident in its ability to lower error rates, surpassing the results of the single model equipped with UMSE. This performance boost is not marginal but statistically significant, suggesting that the dual model is effectively capitalizing on the bifurcation of data to fine-tune the forecast for each segment-underestimated and accurately estimated.

**Table 5:** Results in MAE on evaluating the dual model structure.

**Model**	**Beijing**	**London**	**Taxi**
LightGBM + UMSE	15.431	3.875	5.663
Dual LightGBM + UMSE	13.115^†^	3.449^†^	5.427^†^
GRU + UMSE	13.018	3.429	5.683
GRU-D + UMSE	12.907	3.448	5.721
Dual GRU + UMSE	12.755^†‡^	3.424	5.674^†‡^

^†^Indicates passing *t*-test comparing with baseline model (*p*-value = 0.05).

^‡^Indicates passing *t*-test comparing with GRU-D + UMSE (*p*-value = 0.05).

**Table 6:** Results in RMSE on evaluating the dual model structure.

**Model**	**Beijing**	**London**	**Taxi**
LightGBM + UMSE	30.769	6.326	12.867
Dual LightGBM + UMSE	27.542^†^	5.587^†^	12.622^†^
GRU + UMSE	27.216	5.539	12.817
GRU-D + UMSE	27.102	5.566	12.837
Dual GRU + UMSE	26.718^†‡^	5.522^†‡^	12.788^†‡^

^†^Indicates passing *t*-test comparing with baseline model (*p*-value = 0.05).

^‡^Indicates passing *t*-test comparing with GRU-D + UMSE (*p*-value = 0.05).

These enhancements are not just numerical improvements but represent a substantial advancement in forecasting methodology. The dual model's tailored approach to handling underestimated data points ensures that the forecasting mechanism is more aligned with the true distribution of the datasets. It is not only about reducing the error metrics but also about understanding and addressing the underlying biases in the data more effectively. Statistical significance, as shown by the t-test results, adds robustness to these findings, indicating that the dual model's performance is not only better by measurement but also reliably different from the comparison models. The consistent pattern across different datasets solidifies the dual model's adaptability and its potential as a universally applicable solution for unilateral boundary problems in time series data.

Furthermore, the dual model structure's success in this context underscores the importance of model architecture in handling complex data scenarios. It implies that in cases where data is systematically skewed or underestimated, traditional single models may not be sufficient. A nuanced approach, such as the proposed dual model, which can differentiate and independently process the distinct aspects of the data, can lead to more accurate predictions and a deeper understanding of the data dynamics.

#### 5.2.4 Benefits of feature reconstruction

The experimental results regarding the benefits of Feature Reconstruction (FR) are exhibited in Tables 7, 8. We can find that the integration of FR into the dual LightGBM and dual GRU models demonstrates a clear improvement in forecasting accuracy across various datasets. Feature Reconstruction acts as a key enhancement, refining the predictive capabilities of the dual models. By reconstructing underestimated features, the models can adjust their predictions with greater precision, leading to more accurate forecasts. The dual model's inherent strength in managing underestimated and accurately estimated data points separately is further augmented by FR. The synergy between the dual structure and FR allows for a more nuanced approach, as it provides the model with a more complete picture of the data landscape, enabling it to address the specific challenges posed by underestimation.

**Table 7:** Results in MAE on evaluating feature reconstruction (FR).

**Model**	**Beijing**	**London**	**Taxi**
Dual LightGBM	13.115	3.449	5.427
Dual LightGBM + FR	12.731^†^	3.410^†^	5.361^†^
Dual GRU	12.755	3.423	5.674
Dual GRU + FR	12.783	3.383^†^	5.630^†^

^†^indicates passing *t*-test comparing with the baseline model (*p*-value = 0.05).

**Table 8:** Results in RMSE on evaluating feature reconstruction (FR).

**Model**	**Beijing**	**London**	**Taxi**
Dual LightGBM	27.542	5.587	12.622
Dual LightGBM + FR	27.388^†^	5.537^†^	12.517
Dual GRU	26.718	5.523	12.788
Dual GRU + FR	26.874	5.511^†^	12.767^†^

^†^indicates passing *t*-test comparing with the baseline model (*p*-value = 0.05).

Statistical significance testing further underscores the value of FR. The marked improvements in forecasting accuracy confirm that the inclusion of FR leads to statistically reliable advancements over models without it. The positive impact of FR is evident across different datasets, suggesting a broad applicability of this approach in diverse forecasting scenarios. It's also worth noting that the benefits of FR are not uniform across all error metrics or models, which implies that the effectiveness of FR may depend on the specific characteristics of the dataset and the underlying model dynamics. Nonetheless, the overall trend indicates that FR is a valuable addition to the forecasting process.

#### 5.2.5 Comparative performance against LSTNet

The comparative analysis against LSTNet is displayed in Tables 9, 10. The results reveal that the proposed Unilateral LightGBM and Unilateral GRU models have markedly improved performance. Such an outcome suggests that these unilateral models, tailored to specifically address underestimation in time series data, exhibit a significant advancement over the traditional LSTNet approach. The proposed unilateral models show a clear edge, as indicated by the t-test results, highlighting a statistically significant enhancement in forecasting accuracy. This improvement is notable across different datasets, underlining the robustness of these models in a variety of contexts. The unilateral models' superior performance can be attributed to their specialized dual model structure, the asymmetric loss function, and the reconstruction of features, which are designed to tackle the challenges posed by unilateral underestimation directly.

**Table 9:** Results in MAE on comparing with LSTNet.

**Model**	**Beijing**	**London**	**Taxi**
LSTNet	22.282	4.959	8.058
Unilateral LightGBM	13.795	3.410^†^	5.362^†^
Unilateral GRU	13.083^†^	3.452	5.670

^†^indicates passing *t*-test comparing with the baseline model (*p*-value = 0.05).

**Table 10:** Results in RMSE on comparing with LSTNet.

**Model**	**Beijing**	**London**	**Taxi**
LSTNet	38.723	8.243	16.720
Unilateral LightGBM	28.019	5.537^†^	12.571^†^
Unilateral GRU	26.874^†^	5.511	12.767

^†^indicates passing *t*-test comparing with the baseline model (*p*-value = 0.05).

## 6 Conclusions

In this paper, we addressed the challenge of unilateral boundary time series forecasting, a niche yet critical domain within the broader field of predictive analytics. This issue is particularly relevant in scenarios where time series data are subject to systematic underestimation or overestimation, a common occurrence in diverse applications ranging from environmental monitoring to demand estimation. Recognizing the lacuna in existing literature for models adept at handling such skewed data, our research sought to develop a comprehensive framework explicitly tailored to this unique forecasting challenge.

Our novel approach is multi-pronged, focusing on three integral aspects: the deployment of the unilateral mean square error (UMSE) loss function, the conceptualization of a dual model structure, and the implementation of feature reconstruction. To substantiate the efficacy of our approach, we employed LightGBM and GRU models, renowned for their effectiveness in time series analysis. Through rigorous experimentation across various datasets and underestimation simulations, we demonstrated the model-independence of our approach and its applicability to different forecasting paradigms. Our empirical findings were robust, underscoring the adaptability and potency of our methods in diverse underestimation scenarios.

In short, we have not only proposed a cohesive and generalized model for unilateral boundary time series forecasting but have also empirically validated its superiority over existing methods. This model stands as a significant advancement for real-world applications where the accurate prediction of time series data is vital. Its versatility across different model architectures makes it a promising solution for a wide array of industries, from environmental monitoring to intelligent transportation systems. We believe that our work will serve as a benchmark for future research in this area, offering a robust foundation for the development of more sophisticated and accurate forecasting models.

Future work on unilateral boundary time series forecasting could expand upon our proposed method in several intriguing directions. One area of potential exploration is the application of our framework to a broader range of deep learning architectures, such as Transformers (Vaswani et al., 2017), to evaluate their effectiveness in capturing long-term dependencies within skewed data. Additionally, investigating the integration of external variables that may influence the underestimation or overestimation of data, like economic indicators or environmental factors, could enhance the model's contextual understanding and predictive power. Another promising avenue could involve the use of unsupervised or semi-supervised learning techniques to better handle scenarios with limited labeled data, which is a common challenge in real-world datasets. Lastly, adapting our methods to real-time forecasting systems, where unilateral boundaries are dynamically shifting, could significantly benefit industries requiring instantaneous data analysis and decision-making.

## Data availability statement

The original contributions presented in the study are included in the article/supplementary material, further inquiries can be directed to the corresponding author.

## Author contributions

C-MC: Data curation, Investigation, Methodology, Validation, Writing – original draft. C-TL: Investigation, Methodology, Supervision, Validation, Writing – review & editing. S-DL: Conceptualization, Investigation, Methodology, Project administration, Resources, Supervision, Writing – review & editing.

## Footnotes

^1^ https://github.com/txytju/air-quality-prediction

^2^ https://www.kdd.org/kdd2018/kdd-cup
